# Control of coherent information via on-chip photonic–phononic emitter–receivers

**DOI:** 10.1038/ncomms7427

**Published:** 2015-03-05

**Authors:** Heedeuk Shin, Jonathan A. Cox, Robert Jarecki, Andrew Starbuck, Zheng Wang, Peter T. Rakich

**Affiliations:** 1 Department of Applied Physics, Yale University, New Haven, Connecticut 06520, USA; 2 Sandia National Laboratories, PO Box 5800, Albuquerque, New Mexico 87185, USA; 3 Department of Electrical and Computer Engineering, University of Texas at Austin, Austin, Texas 78758, USA

## Abstract

Rapid progress in integrated photonics has fostered numerous chip-scale sensing, computing and signal processing technologies. However, many crucial filtering and signal delay operations are difficult to perform with all-optical devices. Unlike photons propagating at luminal speeds, GHz-acoustic phonons moving at slower velocities allow information to be stored, filtered and delayed over comparatively smaller length-scales with remarkable fidelity. Hence, controllable and efficient coupling between coherent photons and phonons enables new signal processing technologies that greatly enhance the performance and potential impact of integrated photonics. Here we demonstrate a mechanism for coherent information processing based on travelling-wave photon–phonon transduction, which achieves a phonon emit-and-receive process between distinct nanophotonic waveguides. Using this device, physics—which supports GHz frequencies—we create wavelength-insensitive radiofrequency photonic filters with frequency selectivity, narrow-linewidth and high power-handling in silicon. More generally, this emit-receive concept is the impetus for enabling new signal processing schemes.

## Abstract (web summary)

Integrated photonics hold potential for signal processing but some operations are difficult to perform in all-optical devices. Here, Shin *et al*. use the coupling between coherent photons and phonons in nanophotonic waveguides to demonstrate frequency-selective, narrow-linewidth radiofrequency photonic filters.

## Graphical abstract

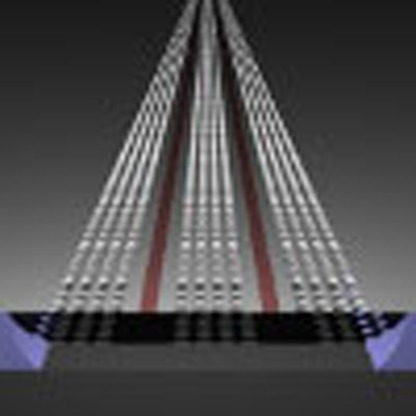

A variety of micro- and nano-scale systems have recently been used to enhance and tailor the interaction between photons and phonons through engineerable structures and materials^1^,^2^,^3^,^4^,^5^,^6^,^7^,^8^,^9^,^10^,^11^,^12^. In the quantum limit, highly controlled photon–phonon couplings permit ground-state cooling of microscopic optomechanical systems^13^,^14^,^15^,^16^, and offer a unique quantum interface between the optical, microwave and phononic domains^17^,^18^. In the classical limit, such photon–phonon interactions are also of significant technological value since they enable new chip-scale radiofrequency (RF) photonic signal processing approaches that combine the merits of both photonic and phononic excitations^3^,^19^,^20^. Specifically, with efficient and reversible conversion between photonic and phononic domains, long coherence time and low-velocity GHz acoustic phonons can be used to slow, store and frequency-shift optical signals^4^,^5^,^10^,^21^,^22^,^23^. By harnessing such interactions, phonons can act as coherent memory^21^,^22^, produce filters with high-frequency selectivity^9^,^24^, and enable a host of processes having no analogue in all-optical photonics^3^,^5^,^20^,^23^,^25^,^26^,^27^. Although engineerable coupling between resonant photonic and phononic modes has been achieved in a variety of chip-scale systems^3^,^4^,^5^,^6^,^13^,^14^,^15^,^16^,^19^,^20^,^28^,^29^,^30^,^31^, a significant challenge to the creation of robust new silicon-based coherent signal processing technologies remains: the realization of narrow-band filters that simultaneously achieve high optical power handling, low signal distortion and wavelength insensitivity^32^,^33^.

We address this challenge through a novel chip-scale multiport photonic–phononic emitter–receiver (PPER) system that produces strong photon–phonon coupling without use of optical resonance. This travelling-wave geometry enables independent control of guided photonic and phononic modes, as well as their interaction. In this system (Fig. 1a,b), forces produced by travelling optical signals transduce coherent phononic signals in the core of an (emitter) optical waveguide (Fig. 1c); the surrounding phononic crystal (PnC) superstructure then shapes the transfer of the phononic signal to an adjacent (receiver) optical waveguide (Fig. 1d), which converts the signal back to the optical domain through photoelastic coupling (Fig. 1e). The PnC superstructure provides tailorability of the phononic transfer function, permitting control of transduction bandwidth, frequency and conversion efficiency at GHz frequencies.

This PnC superstructure (Fig. 1a) produces multi-pole response functions that cannot be created by the singly resonant Brillouin-active membrane (BAM) structures of ref. 34. In contrast to ref. 34, this emitter–receiver system (Fig. 1a) uses PnC line-defects to guide phonons through Bragg reflection; silicon waveguides within each PnC line-defect simultaneously guide light by total internal reflection. The finite reflectivity of the central PnC region (Fig. 1a) permits controllable evanescent coupling between the phononic defect-modes of the emitter- and receiver-ports; hence, coupling-induced mode-hybridization creates guided PnC supermodes of distinct frequencies, yielding a multi-pole phononic transfer function. Such multi-pole transfer functions have frequency selectivities that are far superior to singly resonant systems of ref. 34, an essential feature for high-performance signal processing applications^35^. Beyond the dual-port system of Fig. 1a, this PPER device topology is easily generalized; higher-order (third, fourth, fifth order) responses are produced by increasing the number of coupled line-defects. Hence, this general PPER device concept opens up a compelling new design space for hybrid photonic–phononic signal processing.

To date, much of integrated photonics has relied on optical resonators for RF-photonic filtering operations. However, as the RF signal bandwidths and the optical carriers differ in frequency by many orders of magnitude, narrow band RF filtering becomes a very challenging task. For instance, a second-order filter with 3-MHz bandwidth—comparable in performance to this PPER system—would require two ultra-high Q-factor (~10^8^) optical cavities with precisely controlled cavity frequencies and mode coupling. Nonlinear absorption in silicon severely limits the power levels in such high-Q cavities^36^. Moreover, narrow linewidth lasers must be actively frequency-stabilized relative to the cavity resonances when used as filters. These factors often limit the practicality of narrow-band all-optical filtering operations in RF-photonics. Alternatively, backwards stimulated Brillouin scattering (SBS) has been studied extensively as a means of implementing RF-photonic filters^9^,^24^. However, such backward-SBS filtering schemes have proven difficult to implement in silicon because of weak photon–phonon couplings produced by silicon in the backward-scattering geometry^37^,^38^.

The advantages of this PPER are numerous; this device concept is readily implemented in numerous material systems, spatially separate ports of this system yield negligible optical cross-talk and the emit–receive functionality is inherently wavelength insensitive (see Supplementary Note 1). The optical waves in the emit- and receive-ports can be continuously tuned over a wide wavelength range (>100 nm) with little effect on the transduction efficiency; hence, narrow band filtering and wideband wavelength conversion are simultaneously achieved in this travelling-wave system.

In this paper, we demonstrate phonon-mediated information transfer between the emit- and receive-ports of a dual-channel PPER seen in Fig. 1a. Experiments reveal a highly tailorable two-pole (or second order) transfer function with filter bandwidths as narrow as ~3 MHz, and >70 dB out-of-band signal rejection. Information transfer is largely independent of the wavelength and temporal coherence of the optical waves in the emit- and receive-ports. Applying this concept, we synthesize high-performance 2-port RF-photonic filters; broadband wavelength conversion, RF signal mixing and ultra-narrow-band signal filtering are achieved together with high-optical power handling (>100 mW), a much needed combination in integrated photonics^4^,^32^,^33^. In what follows, we first examine the physics of photon–phonon coupling in a single-channel Brillouin-active waveguide that utilizes Bragg reflection to achieve phonon guidance within PnC defect modes at selected frequencies in the PnC stopband; this system serves as a key building block for wavelength-insensitive second-order RF-photonic filters. Building on this device physics, two such PnC defect states are evanescently coupled to produce the travelling-wave PPER system seen in Fig. 1a. Adjusting the device geometry, the PnC supermodes of this system can be engineered to shape the signal transfer response of the emitter–receiver pair. In addition, the underlying physics of this PPER system is accurately captured using coupled mode theory; hence, by straightforward extension of such models, one can readily explore the response of more complex PPER systems and we identify the key factors that impact filter shape and transduction efficiency.

## Result

### Brillouin-active PnC waveguide

**Figure 1:**
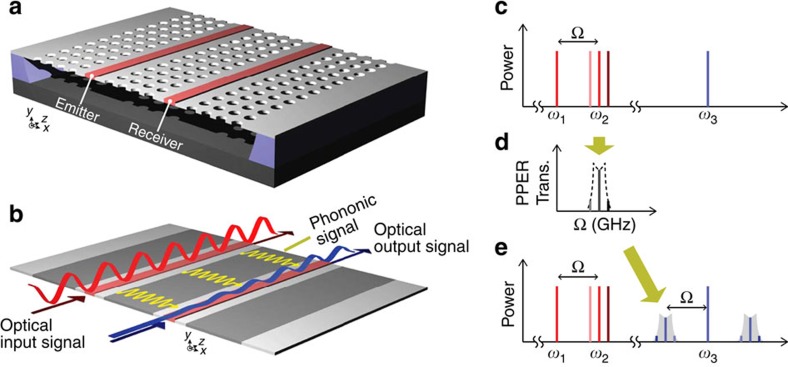
Travelling-wave photonic–phononic emitter–receiver (PPER). (**a**) Schematic of a PPER system consisting of two silicon optical waveguides (red) embedded in a phononic crystal membrane (grey). (**b**) Diagram showing principle of PPER operation. Red, blue and yellow curves are the optical input signal, optical output signal and transduced phonon waves, respectively. Information is encoded on the red wave (emitter) through amplitude modulation; transduced phonons then couple this information to a monochromatic blue wave (receiver) of disparate wavelength via parametric coupling. (**c**–**e**) Characteristic spectra showing the input (**c**) and output (**e**) optical signals and the response produced by phononic supermodes that mediate information transfer (**d**). In the emitter port, a pump field (*ω*_2_=*ω*_1_+Ω) is swept relative to a local oscillator (*ω*_1_) to produce an amplitude modulated beat-note. Optical forces generated in the emitter waveguide drive the excitation of phonons. Information is transferred between the emitter and receiver ports by phonon supermodes that produce the transfer function in **d** (black dashed). Information is encoded on the blue probe field (*ω*_3_) of the receiver port for frequencies within the transfer function of the phononic supermodes (shaded grey in **e**).

**Figure 2:**
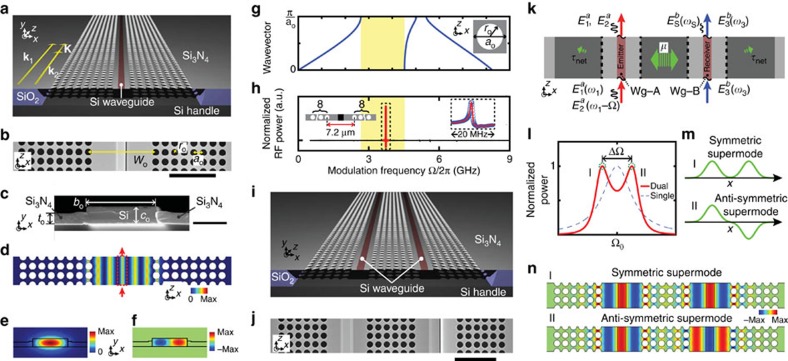
Brillouin-active membrane waveguide embedded in phononic crystal structures. (**a**) Schematic showing the anatomy of PnC-BAM waveguide with the wavevectors of the optical pump (**k**_1_), the scattered light (**k**_2_) and the phonon (**K**). (**b**) Top-down scanning electron microscopy (SEM) image of a segment of the PnC-BAM waveguide. The defect size of the PnC structure, *W*_o_, is defined as the centre-to-centre distance between inclusions on either side of silicon waveguide. The lattice constant, *a*_o_, is 1 μm , and the radius of holes, *r*_o_, was 0.385a_o_. Scale bar, 5 μm. (**c**) SEM image of the cross-section of the waveguide core within the nitride membrane. The size (*b*_o_ × *c*_o_) of the silicon waveguide is 950 × 220 nm^2^, and the thickness (*t*_o_) of silicon nitride is 130 nm. Scale bar, 0.5 μm. (**d**) Computed acoustic energy density in a unit cell of PnC-BAM waveguide at a resonant frequency of 3.72 GHz. The red dotted box indicates the position silicon waveguide core. (**e**) Computed *E*_x_ fields of the optical mode in a silicon waveguide. (**f**) Computed *x* component of the electrostrictive force density from the guided optical mode. (**g**) Dispersion diagram of the 2D phononic crystal cladding (unit cell shown in the inset) showing phonon frequency versus *X*-direction phonon wave vector. (**h**) Spectrum of Brillouin responses obtained through heterodyne four-wave mixing measurements. The output signal from the PnC-BAM waveguide for *W*_o_=7.2 μm is normalized to that of a reference silicon waveguide (Brillouin inactive) under identical experimental conditions. The left inset is the schematic geometry of the PnC-BAM waveguide. (**i**) Schematic showing dual-channel PnC-BAM waveguide that forms the PPER system under study. (**j**) Top-down SEM image of a portion of the dual-channel PPER system. Scale bar, 5 μm. (**k**) Pictorial representation of PPER systems. *μ* and *τ*_net_ represent the coupling rate between adjacent PnC defect modes and net phonon decay rate, respectively. (**l**) Sketch showing the parametric responses for single (blue dashed) and dual (red solid) channel PnC-BAM waveguides. (**m**) Illustration of the displacement distribution for symmetric (I) and anti-symmetric (II) supermodes. (**n**) Simulated elastic displacement fields (*x*-displacement) associated with symmetric and antisymmetric supermodes of the PPER system.

We first examine the strength of travelling-wave photon–phonon transduction generated by a stand-alone silicon waveguide embedded within a silicon nitride membrane (see Fig. 2a). This structure guides both optical photons and acoustic phonons, whereas the photoelastic response of the silicon waveguide core mediates reciprocal photon–phonon coupling. As the active component of the emitter–receiver pair seen in Fig. 1, the transduction efficiency of this system is of critical importance. The highly tensile silicon nitride membrane is patterned to form two PnC regions, consisting of a square lattice of holes, placed symmetrically about the silicon waveguide core. Bragg reflection produced by these PnC regions creates guided phonon modes (that is, defect modes) in the phononic stopband. The silicon waveguide core centred within the phononic waveguide yields tight confinement of the guided optical mode through total internal reflection. A top-down scanning electron microscopy image of the fabricated BAM waveguide with a surrounding PnC structure (PnC-BAM waveguide) is seen in Fig. 2b along with a magnified view of the waveguide cross-section (950 × 220 nm^2^) in Fig. 2c. Throughout this article, the lattice constant, *a*_o_, is 1 μm, and the radius of holes, *r*_o_, is 0.385 μm. In comparison to a related structure^34^, the enlarged silicon core of this waveguide results in lower propagation loss (<1 dB cm^−1^), lower nonlinear absorption^39^, and higher power handling (>110 mW), which contribute to significant performance enhancements. For further details about power handling, see Supplementary Note 2.

Strong coupling between these co-located optical and phononic modes is mediated by optical forces generated within the silicon waveguide core^37^,^40^. This form of travelling-wave photon–phonon coupling is termed forward-SBS or stimulated Raman-like scattering^41^,^42^. Through forward-SBS, energy can be transferred between optical pump and signal waves propagating within the waveguide. Full-vectorial multi-physics simulations^34^,^38^,^40^ were used to compute photon–phonon coupling within a PnC-BAM waveguide of dimension *W*_o_=7.2 μm, and reveal that the PnC defect mode of Fig. 2d is efficiently excited at a frequency of 3.72 GHz within the stopband in Fig. 2g. Figure 2e,f shows the computed *E*_*x*_-field of the fundamental transverse-electric (TE)-like mode of the silicon waveguide and the *x*-component of the corresponding electrostrictive force density, respectively. A vector phase matching diagram for forward-SBS (yellow arrows) is shown in Fig. 2a. Here, a phonon of wave-vector **K**=**k**_1_−**k**_2_ and frequency Ω=*ω*_1_−*ω*_2_ mediates this interaction, where **k**_j_ and *ω*_j_ are the wave-vector and frequency of interacting optical waves. Phase matching requires that the group velocity of the optical signal match the phase velocity of the guided phonon mode (Ω/|**K**|). This condition is only satisfied by guided phonon modes^34^ with ultra-slow group velocity (∂Ω/∂|**K**|~1 m s^−1^). For further discussion of phase-matching in such forward-scattering geometries, see the subsection entitled ‘Phase-matched coupling via forward SBS’ in ref. 34.

In these silicon waveguides, photon–phonon coupling is predominantly mediated by electrostrictive forces as the contribution to SBS nonlinearities produced by radiation pressure decreases rapidly with increasing waveguide size^38^,^40^. The optical force distribution of the fundamental TE-like mode (see Fig. 2f) produces strong coupling to symmetric zero-order Lamb-waves. Hence, the excited PnC defect mode is a symmetric Lamb-wave with a small flexural character because of the vertical asymmetry of the protruding silicon waveguide core, as seen in Fig. 2c. (For more information on the character of the guided membrane modes, see the Supplementary Information of ref. 34.) The computed phononic dispersion curves associated with the symmetric Lamb waves within the fabricated 2D PnC structure are seen in Fig. 2g^43^. Note that defect-mode confinement does not necessitate a complete phononic bandgap as the wave-vector of the emitted phonons is nearly perpendicular (100 microradians from normal) to the direction of light propagation. Moreover, the phase matching conditions of forward-SBS, seen in Fig. 2a, only permit strong optical coupling to phonons with wave-vectors in the ***Γ***−***X*** direction. Hence, only the dispersion in the ***Γ***−***X*** direction, seen in Fig. 2g, is relevant to the creation of Brillouin-active defect modes. As seen from the highlighted (yellow) region of Fig. 2g, the fundamental phononic stopband associated with the Brillouin-active symmetric Lamb-waves extends from 2.6 to 4.5 GHz. By designing the dimension of the phononic defect, *W*_o_, individual Brillouin-active PnC defect modes can be created at select frequencies within this stopband, yielding strong Brillouin resonances and strong photon–phonon coupling. Here, the synthesis of isolated Brillouin resonances contrasts with the structures of ref. 34, which are seen to produce a series of acoustic overtones.

The photon–phonon coupling was quantified through studies of the fabricated PnC-BAM waveguide (Fig. 2b,c), which is suspended continuously over a 4-mm length. Lithographically tapered silicon input and output couplers ensure that only the fundamental TE-like mode (seen in Fig. 2e) is excited. The strength of photon–phonon coupling was experimentally determined through heterodyne four-wave mixing experiments^34^, enabling the study of Brillouin-active phonon modes between 0.5 and 9 GHz. The dashed inset of Fig. 2h shows the result of one such four-wave mixing measurement; coherent interference between the Kerr and Brillouin nonlinear susceptibilities produces a characteristic Fano-like line-shape. The analysis of this line-shape provides a measurement of Brillouin nonlinearity relative to the intrinsic Kerr nonlinearity^34^, yielding a Brillouin gain coefficient of *G*_SBS_=2|γ_SBS_|=1,960±355 W^−1^ m^−1^ and a linewidth of 1.2 MHz (or Q-factor of ~3,160 at a centre frequency of 3.72 GHz).

### Physics of the phonon emitter–receiver

In addition, such PnC geometries provide control of phonon emission and coupling as the basis for new travelling-wave phonon emit–receive functionalities. The anatomy of a travelling-wave phonon emitter–receiver pair—in the form of a dual PnC-BAM waveguide—is shown in Fig. 2i for comparison with the top-down scanning electron microscopy image of Fig. 2j. This system is comprised of two silicon waveguides embedded within a silicon nitride PnC superstructure having two PnC line-defects (*W*_o_=5.7 μm). The *E*_*x*_-field of the fundamental TE-like mode of each silicon waveguide is identical to that in Fig. 2e. Each PnC line-defect is bounded by symmetrically placed PnC regions (*N* periods each), whereas a centrally located PnC region (*N*_c_ periods) separates the line defects by a centre-to-centre distance of [(*N*_c_−1) × *a*_o_+*W*_o_]. Figure 2i,j shows the special case of *N*=*N*_c_=6. Silicon waveguides are centred in each line-defect (as seen in Fig. 2j), producing a dual channel system with mirror symmetry.

The diagram of Fig. 2k illustrates the basic physical principle by which the phonon emit–receive geometry transfers information from light within emitter waveguide (Wg-A) to light in receiver waveguide (Wg-B). Through this reciprocal process, electrostrictive optical forces produced by optical signals within Wg-A drive the excitation of coherent phonons; the surrounding PnC superstructure then shapes the transfer of phononic information between Wg-A and Wg-B. Through this process, phononic energy transfer is mediated by PnC supermodes consisting of evanescently coupled PnC defect modes within the superstructure (as illustrated by Fig. 2m). The transduced phononic information is then encoded on optical waves carried by Wg-B through photoelastic coupling. In contrast to the phononic properties of the system, negligible optical cross-talk occurs between Wg-A and Wg-B as the guided optical modes decay rapidly (~60 nm) outside of the silicon waveguide core (as seen in Fig. 2e). This guarantees that the signal transduction occurs only through the phononic domain, yielding low background noise level and high dynamic range. The coupling rate (*μ*) between the phononic defect modes is mediated by the central PnC coupling region (of *N*_*c*_ periods), whereas the external decay rate 
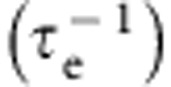
 from each defect mode is determined by the PnC cladding region (of *N* periods) on either side of the device.

The spatial dynamics of such phonon-mediated coupling between the optical waves can be treated analytically using temporal coupled-mode theory^35^,^44^ (see Supplementary Note 3). As illustrated by Fig. 2k, optical fields 
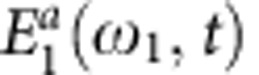
, 
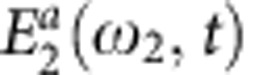
 and 
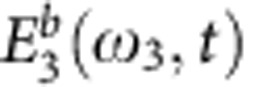
 are injected into the system, and we seek the parametrically generated signal 
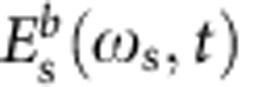
 at the output of Wg-B. Optical forces produced by the interference between 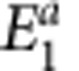 and 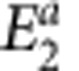 drive the phonon supermodes; these can be expressed as a linear combination of the elastic displacement fields *e*_*a*_(*x*,*y*) and *e*_*b*_(*x*,*y*), representing the PnC defect modes in Wg-A and Wg-B, respectively. The supermodes seen in Fig. 2n show the *x*-displacement associated with symmetric and antisymmetric supermodes. The modal hybridization and the resonant transduction between the Brillouin active modes are accurately captured in terms of the modal coupling rate (*μ*) and the net modal decay rate 
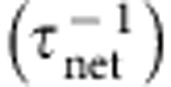
. Using optical forces to source the excitation of the PnC defect mode in Wg-A, modal perturbation theory can be used to determine the parametric growth of 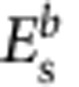 in Wg-B. The growth rate of the signal wave amplitude, *B*_s_, in the *z*-direction of propagation is,


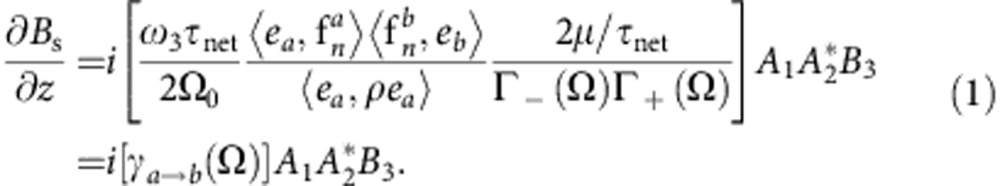


Here, γ_*a*→*b*_(Ω) represents the phonon-mediated coherent coupling from Wg-A to Wg-B, and Γ_±_(Ω)≡[Ω−(Ω_0_±*μ*)+*i*/*τ*_net_]. We use the following definitions: Ω_0_ is the natural frequency of uncoupled phonon modes; *ρ*(*x*,*y*) elastic medium mass density; 
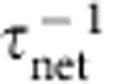
, 
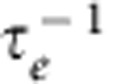
 and 
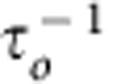
 are the net, external and internal phonon decay rates, where 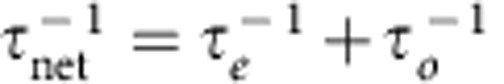; 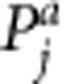 and 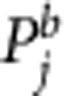 are the powers carried by 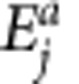 and 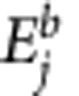; *A*_*j*_ and *B*_*j*_ are the normalized wave amplitudes of 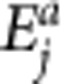 and 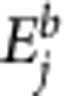 such that 
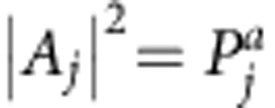
 and 
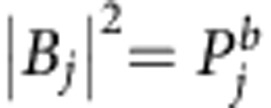
; and 
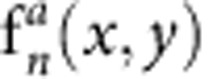
 and 
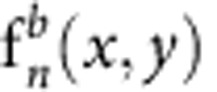
 are the power normalized force densities produced by light in Wg-A and Wg-B under continuous-wave excitation.

As seen in Fig. 2l, phonon-mediated coherent coupling from Wg-A to Wg-B, or γ_*a*→*b*_(Ω), exhibits a sharp second-order response with poles at 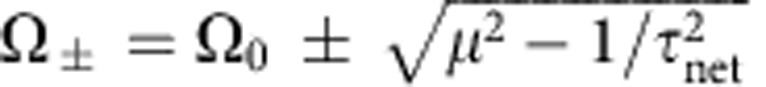. These resonances correspond to symmetric and anti-symmetric phononic supermodes as seen in Fig. 2m,n. Note the second-order response produced by this doubly resonant system has far sharper roll-off than the first-order (Lorentzian) response of single channel systems, as illustrated in Fig. 2l. Owing to the symmetry of this geometry, the elastic displacement field (*e*_*j*_), the power-normalized force density 
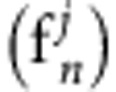
 and the overlap 
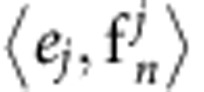
 are effectively identical in both waveguides (*j*=*a*, *b*). As a consequence, |γ_a→b_(Ω_±_)|=*G*_*o*_/2, where *G*_*o*_ is the single-waveguide Brillouin gain (of ref. 38) in the limit as 
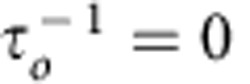
.

In the limiting case when *μ*=0 (*N*_*c*_=∞), we see that no information can be transduced from Wg-A to Wg-B, and the phononic eigenmodes of these waveguides are degenerated (see the dashed curve of Fig. 2l). However, for finite couplings (*μ*>0), hybridization of the Brillouin-active phonon modes produce both symmetric and anti-symmetric supermodes (see Fig. 2m,n) with resonant frequencies (or poles) Ω_+_ and Ω_−_, respectively. These hybridized phonon modes mediate the transfer of coherent information between the two optical waveguides. In experiments, we explore the low gain regime, where the signal power is given by 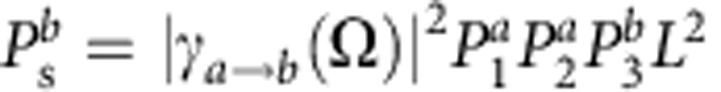. As a result, the power of the signal wave 
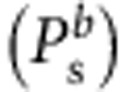
 generated over a length, *L*, increases quadratically with pump power 
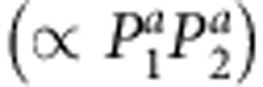
 and length (∝*L*^2^). Note that the concise analytical model (summarized by equation 1) describes the physics of this multi-port PPER system with a few basic physical parameters, greatly reducing the complexity of this nontrivial 3D system. Most crucially, this model provides a means of understanding spectral response of the PPER system and its overall efficiency (as discussed above).

### Demonstration of a phonon emitter-receiver

**Figure 3:**
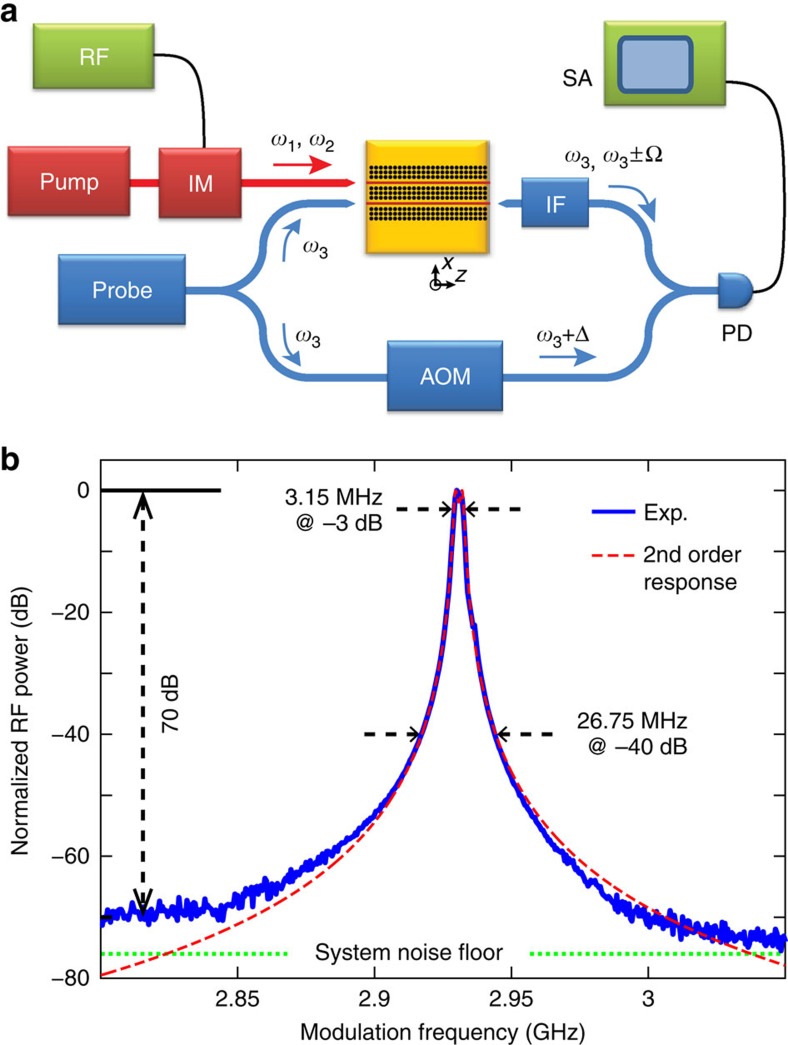
RF photonic response of a dual channel PnC-BAM waveguide. (**a**) Schematic diagram of the apparatus used to measure the Brillouin nonlinearities of PnC-BAM waveguides. Pump: 1,547 nm laser, Probe: 1,536 nm laser, IM: intensity modulator, RF: RF generator for the intensity modulator, AOM: acousto-optical modulator, IF: interference filter, PD: fast speed photodiode receivers, SA: spectrum analyser. (**b**) Normalized RF response of a PPER system. The theoretical prediction with second-order response (red dashed) is atop the experimental data (blue). System noise floor is expressed as a dotted green line.

The photonic–phononic emit–receive functionality is demonstrated using the fabricated dual channel PPER of Fig. 2j. Through experiment, a single-wavelength (1547, nm) laser-line is intensity-modulated to synthesize pump-waves, corresponding to 
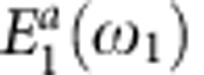
 and 
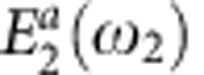
 in Fig. 2k. These waves interfere to drive the excitation of hybridized phonon supermodes, transferring the coherent beat-signal from Wg-A to Wg-B. These phonon supermodes generate a new signal field, 
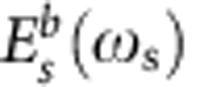
, through travelling-wave phase modulation of the injected probe wave, 
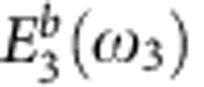
, at a distinct (1536, nm) wavelength. In this way, the beat-signal is coherently transferred from Wg-A to Wg-B. The transduced signal is then measured at the output of Wg-B through heterodyne detection, as shown in Fig. 3a. As Wg-A and Wg-B are optically decoupled, optical crosstalk is negligible. As seen in Fig. 3a, the frequency of excited phonon is controlled by changing the RF modulation frequency (Ω). Sweeping the modulation frequency enables quantitative study of the frequency response of the dual-channel PPER system from 1–9 GHz. (Beyond such frequency response measurements, this PPER system can be used to process coherent signals of arbitrary form by treating 
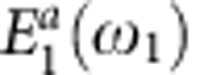
 as a ‘local oscillator’ and encoding data on 
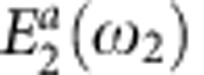
 using phase modulation.)

The measured response of a fabricated emitter–receiver (*W*_o_=5.7 μm, *N*=*N*_*c*_=6), with a 7-mm interaction length, is seen in Fig. 3b. A sharp second-order frequency response is centred at 2.93 GHz (blue), demonstrating efficient phonon-mediated information transfer between Wg-A and Wg-B. The measured data (blue) show a full-width at half-maximum of 3.15 MHz, corresponding to an aggregate Q-factor of ~930. Using a least-squares fit of the theoretical second-order transfer function from equation 1, the parameters showing spectral response of the PPER system are extracted as *μ*=8 MHz, 1*/τ*_net_=6 MHz, and *Q*_net_=*Ω*_*o*_*τ*_net_/2~1,530. The high net Q-factor suggests remarkable structural homogeneity and low phonon dissipation over the entire device length. This sharp second-order response is highly desirable as it yields high selectivity against unwanted signals (or noise). Moreover, building on such device topologies, a variety of higher-order responses can be created using cascaded structures^35^,^45^.

As seen in Fig. 3b, the measured second-order response (blue) shows a tremendous out-of-band rejection of >70 dB; optical crosstalk poses no limitation to the dynamic range of the measured frequency response. The contrast of these measurements is limited only by our measurement noise floor (green dotted). Hence, to an excellent degree, information is transduced between Wg-A and Wg-B solely by the engineered phonon supermodes of the system. Through the measurements seen in Fig. 3, fibre-to-waveguide coupling efficiency limits the pump wave powers 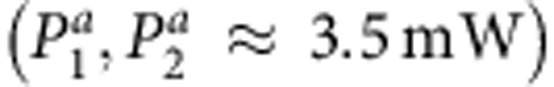 in device, yielding a peak signal conversion efficiency 
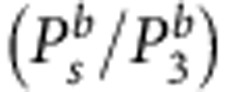
 of ~10^−4^. Note that this efficiency can be significantly enhanced by increasing pump power 
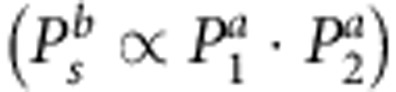
, interaction length 
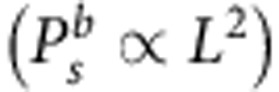
 and Brillouin gain 
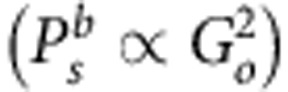
. For instance, with pump powers of 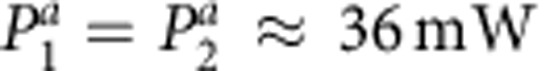, we have achieved the signal conversion efficiency of ~1%. See Supplementary Note 4 for the response spectra demonstrating enhanced signal conversion efficiency. Moreover, efficiencies approaching unity are readily achievable with longer interaction lengths through modified geometries that enhance the photon–phonon coupling strength (*G*_o_) of travelling-wave phonon emit–receive structures.

From these measurements, we demonstrate a wavelength-insensitive transfer function with an unrivalled combination of stopband attenuation (>70 dB), selectivity (second-order response with high slope of 20/3.55(dB MHz^−1^)), linewidth (~3 MHz) and power-handling (>110 mW) in silicon photonics. For comparison, coupled microring cavities have been used to create high-order bandpass filters of ~1 GHz bandwidth with high selectivity^45^. However, intra-cavity power enhancement, combined with the high nonlinearities of silicon, severely limit the power-handling through such all-optical filtering schemes^36^. These characteristics (wavelength-insensitive, power handling, stopband rejection, selectivity, and linewidth) are essential for many high-performance RF photonic applications^32^,^33^,^46^.

Viewing this system as an RF-photonic filtering technology, we see that the PPER pair has a desirable shape factor (8.5), as well as remarkably high slope (~20/3.55(dB MHz^−1^))^47^. (The shape factor is the ratio of full width at −40 dB to full width at −3 dB.) Moreover, the rejection of >65 dB was observed over 1.9-GHz stopband (2.6–4.5 GHz). See Supplementary Note 5 for the response spectra over stopband range. Note that all of these characteristics are controllable by engineering the PnC structure. The physics of the PPER system can readily support operation at >10 GHz as strong photon–phonon interactions at higher frequencies (~18 GHz) have been observed in smaller core BAM waveguides^34^ and the geometry of PnC structures can be engineered to have stopbands at high frequency^43^. Furthermore, using this dual port PPER system, we have also demonstrated wavelength conversion. RF signals encoded on the pump light (in the emit-port) at 1,547 nm are transferred to the receive-port at 1,536 nm through coherent phonon-mediated information transfer.

### Phononic supermode engineering

**Figure 4:**
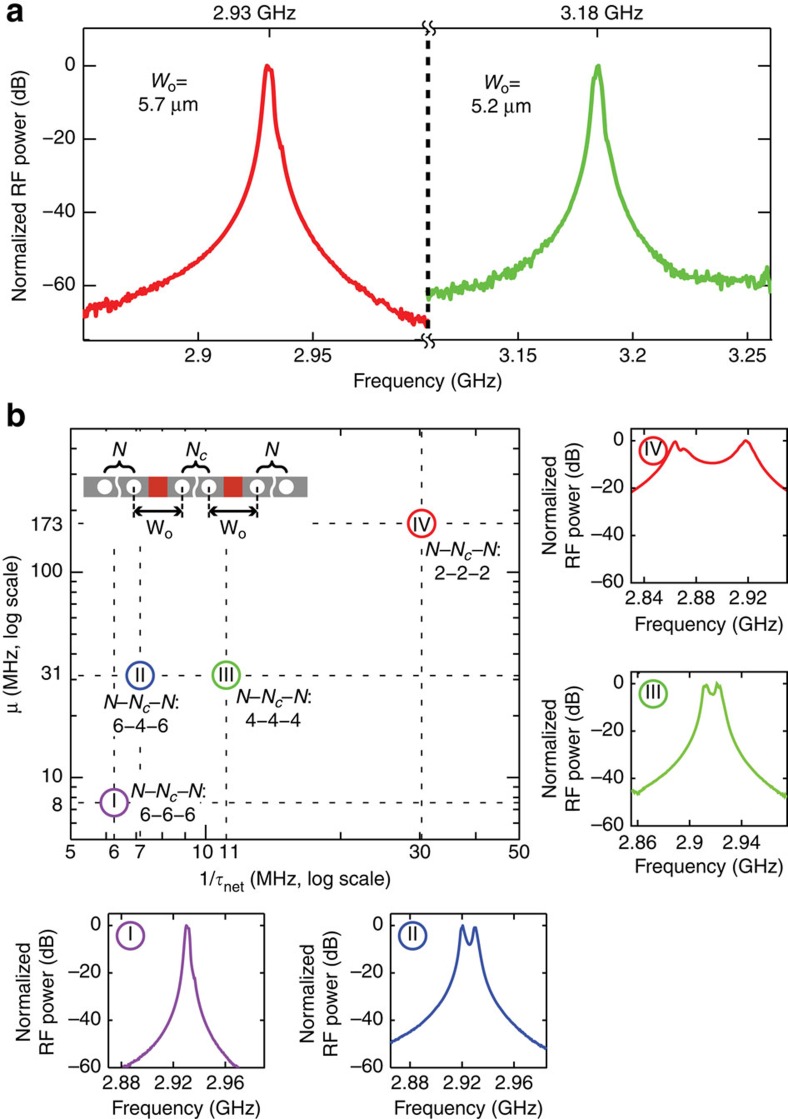
Engineered response of dual-channel PPER. (**a**) Measured RF responses of PPER systems for *W*_o_=5.7 μm (red) and *W*_o_=5.2 μm (green) versus frequency. (**b**) Coupling rates (*μ*) versus net decay rates 
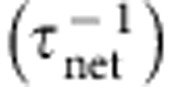
 extracted from measured RF responses (I, II, III and IV) of PPER systems with *N*−*N*_c_−*N*=(6–6–6, 6–4–6, 4–4–4 and 2–2–2), respectively. Results are shown in log–log scale. The inset in the top-left corner is schematic geometry of the BAM waveguides. The insets in the right and bottom corners are the normalized RF power responses (I, II, III and IV) corresponding to the combinations of the PnC layer numbers of *N*−*N*_*c*_−*N*=(6–6–6, 6–4–6, 4–4–4 and 2–2–2), respectively.

As described above, the PPER response is determined solely by phonon supermodes straddling both waveguides. These supermodes are controllable by engineering the PnC structure. Here, we experimentally demonstrate the ability to engineer the response of phonon emitter–receiver systems. Note that the centre frequencies of PPER supermodes can be tailored by engineering the defect size *W*_o_. In Fig. 4a, the emit–receive responses for two different defect sizes are shown for the number of PnC layers, *N*=*N*_c_=6. Although the lineshape does not change significantly, the centre frequency is shifted by 250 MHz as the defect size is lithographically varied from *W*_o_=5.7 μm (red) to 5.2 μm (green). Hence, we can readily tailor the centre frequency of information transduction while preserving the filter shape.

In addition, the PPER transfer function can be shaped by lithographically controlling frequency splitting and decay rate of the PnC supermodes. Hence, phononic supermodes can be engineered by varying, *N*, *N*_c_ and *W*_o_. To demonstrate this control, we examined the RF response of PPER systems with a fixed *W*_o_ of 5.7 μm, whereas lithographically varying *N* and *N*_c_ numbers. Experimentally measured responses for PPER devices with *N*−*N*_c_−*N* values of (6–6–6, 6–4–6, 4–4–4 and 2–2–2) are seen in Fig. 4. From these data, the coupling rate (*μ*) and the net decay rate (1/*τ*_net_) are extracted by fitting the analytical response function (equation 1) to the experimental data of RF response of each device (insets of Fig. 4b). As seen in Fig. 4b, the coupling rate and the net decay rate can be tailored over a significant range by varying the numbers of the PnC coupling layers, *N*_c_, and the PnC cladding layers, *N*, respectively. Note that the phononic coupling rate between Wg-A and Wg-B increases with smaller *N*_c_ values, whereas the phonon lifetime drops with smaller *N* values. Hence, this topology provides unprecedented control over the centre frequency, bandwidth, shape-factor and slope.

## Discussion

In this study, we have demonstrated the travelling-wave PPER and studied the frequency response of coherent information transduction through such device physics. As an RF filter, this PPER system simultaneously possesses high dynamic range (70 dB), high Q-factor, wide rejection bandwidth (~1.9 GHz) and high selectivity (bandwidth of 3 MHz, low shape factor of 5 and slope of 20/3(dB MHz^−1^)). The underlying phonon-mediated mechanism could form the basis for a host of powerful new coherent information processing technologies involving wavelength conversion, amplifier, RF mixing and RF photonic filter. Although we have limited our demonstration to classical signal processing in this paper, with vastly reduced phononic dissipation and thermal noise at cryogenic temperatures, one could envision using such travelling-wave signal processing schemes at the single-photon level with negligible optical crosstalk, enabling forms of single-photon frequency conversion^48^ and quantum information processing^49^.

More generally, this compound emit–receive system behaves as a two-port optical system with negligible optical cross-talk and back-reflection: information is transferred from one port to another through phononic information transduction. As this travelling-wave (or reflectionless) geometry negates the need for optical isolators, this platform is directly compatible with silicon-photonic systems. In addition, counter-propagating modulated pump and probe beams will experience appreciable phase walk-off with the longer length of the PPER system, meaning that this filter operation has directional preference through interband transitions^50^. Building on this concept, non-reciprocal isolator with narrow band schemes in the PPER system akin to refs. 51, 52 can be useful. Moreover, the relaxed phase-matching conditions of this forward-scattering geometry enable narrow-band phononic information transfer (or filtering) in a wavelength-independent manner^34^. As a corollary, the wavelength and even spectral bandwidth of signals in the two input ports can vary drastically, with little or no impact on the information transduction performance. This spectral insensitivity is a tremendous asset for practical applications as high-quality lasers are no longer essential for many RF-photonic applications.

Such wavelength insensitivity, combined with the high-power handling and dynamic range, contrasts sharply with the properties of widely studied resonant cavity-based systems for narrow-band signal processing in silicon^4^,^36^,^46^,^47^,^53^,^54^,^55^. In addition, this hybrid photonic–phononic emit–receive approach yields filter shapes and frequency that unaltered as optical power is varied by orders of magnitude. This approach negates the need for frequency stabilization (or frequency locking), which often limits the practical utility of resonant optical filtering^4^,^36^,^46^,^47^,^53^,^54^,^55^. More generally, as this emit–receive concept opens up a large design space for hybrid photonic–phononic design, it could be the basis for numerous powerful new signal processing schemes.

## Methods

### Fabrication methods

The details of the fabrication process is described in ref. 34, and here we sketch out the process briefly as follows: An ASML deep ultraviolet scanner was used to pattern the silicon cores of the PnC-BAM waveguides on a silicon-on-insulator wafer. After etching the pattern in an AMAT DPS etch tool, a 300-nm Si_3_N_4_ layer was deposited. A chemical-mechanical polish and hot phosphoric acid etch were used to clear the protruding nitride atop the silicon cores. The PnC structures were then patterned on the nitride layer of the wafer, and then etched. The oxide under-cladding was then removed with a 49% HF etch.

### Experimental methods

The pump (1,547 nm) beam is modulated using a Mach-Zehnder intensity modulator; the carrier-frequency component was suppressed by optimizing the bias voltage. The probe (1,536 nm) beam split into two paths, where the upper path interrogates the device under test (DUT) and the lower path serves as the local oscillator. The probe beam in the upper path and the pump beam are coupled separately into the two channels of the DUT using two lensed fibres. The probe beam is then coupled out of the DUT using a lensed fibre, where stray pump light (if any) is filtered out with an interference filter. The probe beam in lower path passes through an acousto-optic modulator with a frequency shift (Δ/2π) of 40 MHz, and is combined with the probe beam in the upper path using a directional coupler. The beat note between local oscillator (*ω*_3_+Δ) and the scattered light from the DUT (***ω***_**3**_±Ω) is recorded using a high-speed photodetector, and is analysed with an RF spectrum analyser. An estimated fibre-to-chip coupling loss of 15 dB and waveguide propagation loss of <1 dB cm^−1^ were found through waveguide cutback measurements. The internal powers of pump and probe beams are 7 and 6.3 mW, respectively.

## Author contributions

All authors made important contributions to this work. R.J. and A.S. fabricated the waveguide devices. P.T.R. designed devices with assistance of J.A.C., H.S. and Z.W. H.S. and P.T.R. performed numerous device simulations to understand various aspects of experiments. H.S. and P.T.R. developed analytical models to describe device performance. H.S. and P.T.R. developed the experimental techniques. H.S. conducted experiments with assistance of P.T.R. H.S. and P.T.R. conceived and designed the experiments. All authors contributed to the writing of this paper.

## Additional information

**How to cite this article:** Shin, H. *et al*. Control of coherent information via on-chip photonic–phononic emitter–receivers. *Nat. Commun.* 6:6427 doi: 10.1038/ncomms7427 (2015).

## Supplementary Material

Supplementary Information Supplementary Figures 1-3, Supplementary Notes 1-5 and Supplementary References
