# Supplementary material for: Control of coherent information via on-chip photonic–phononic emitter–receivers
Source: Nat Commun. 2015 Mar 5;6:6427. doi: 10.1038/ncomms7427 (PMC4366499; doi:10.1038/ncomms7427)
Supplement: Supplementary Information — Supplementary Figures 1-3, Supplementary Notes 1-5 and Supplementary References [file ncomms7427-s1.pdf]

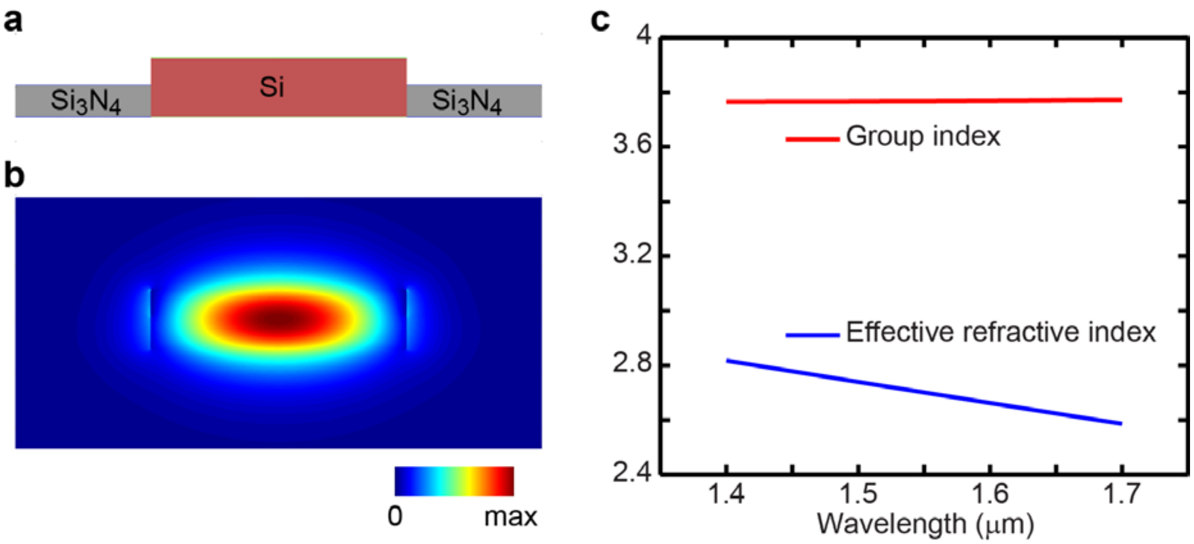

Supplementary Figure 1| Group index and effective refractive index of the optical waveguides. a, The waveguide geometry used in the simulation. The silicon waveguide core has the size of $950 \times 220\ \mathrm{nm}^2$, and the thickness of silicon nitride membrane is 130 nm. **b,** Computed E_x fields of the guided optical mode in the silicon waveguide. **c,** Computationally calculated group index and effective refractive index of the waveguide.

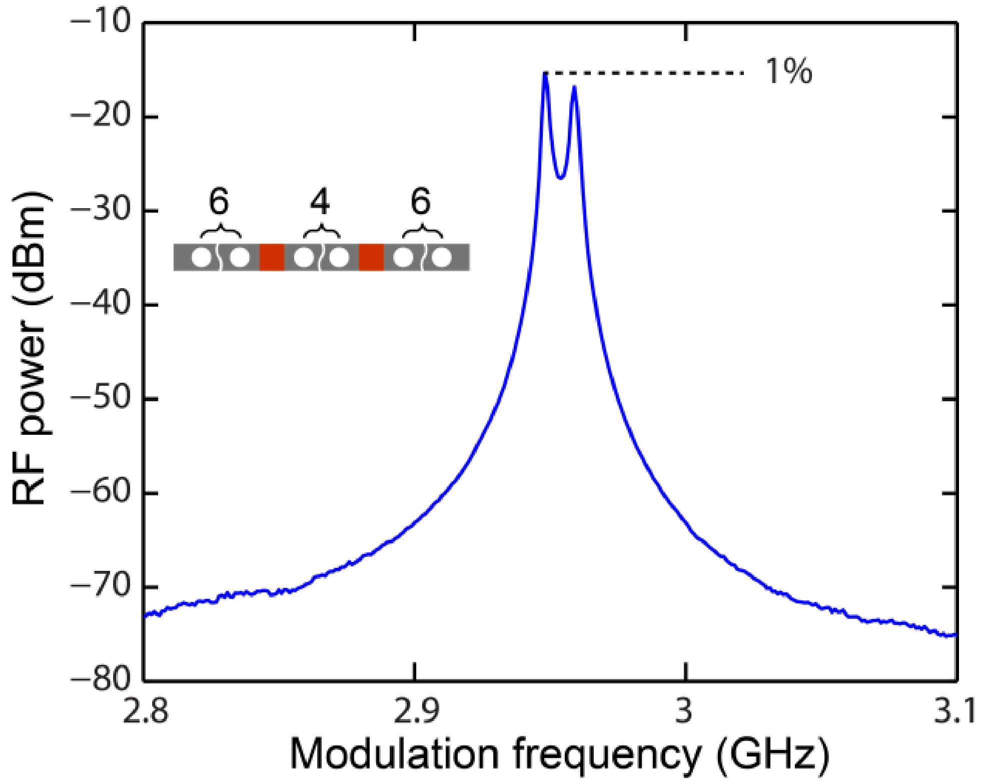

Supplementary Figure 2| Spectrum of photon-phonon emitter-receiver responses. The measured RF power spectrum is displayed through the dual channel PnC-BAM waveguides for $W_o = 5.7\ \mu m$ and $N - N_c - N$ = 6 – 4 – 6.

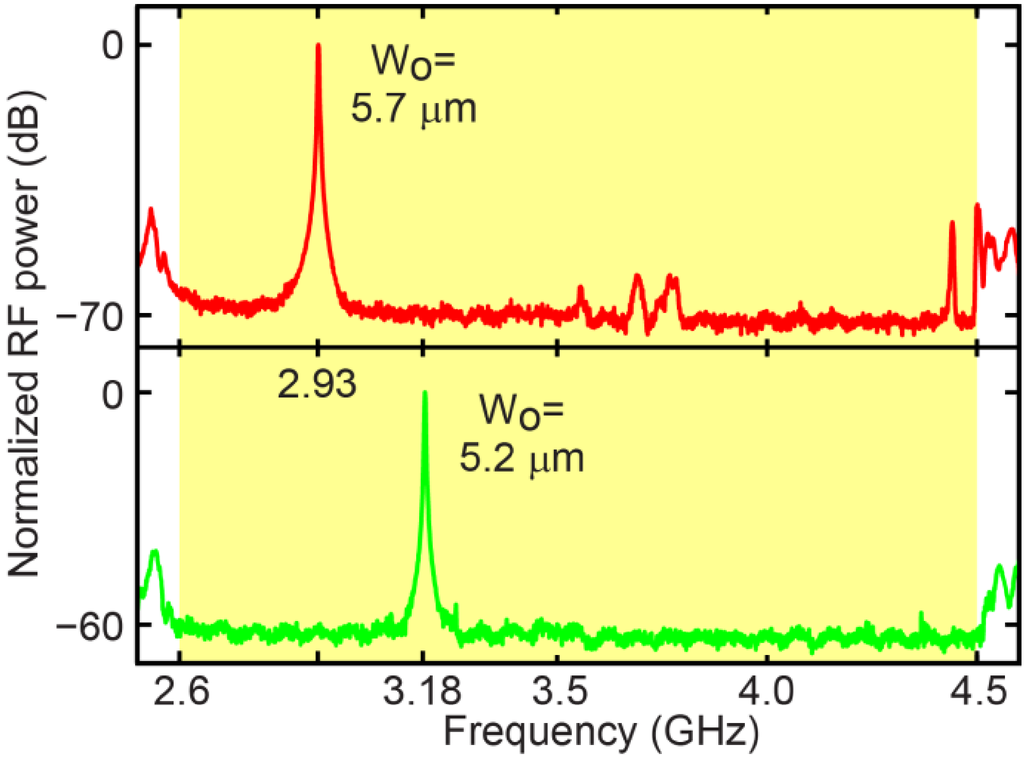

Supplementary Figure 3| Spectra of photon-phonon emitter-receiver functionalities over the stopband range. The normalized RF responses produced by the signal field are displayed through the dual channel PnC-BAM waveguides for $W_o = 5.7\ \mu m$ (red) and $W_o = 5.2\ \mu m$ (green). The highlighted (yellow) region represents the fundamental phononic stopband (2.6 – 4.5 GHz).

Supplementary Note 1

Wavelength and laser linewidth insensitivity.

The generation and detection of coherent phononic waves are proportional to the magnitude of the optical power modulation[1]. This indicates that laser linewidth of the waves in either optical waveguides does not limit the performance of the PPER system. For instance, the laser linewidth of the probe beam under our experimental conditions is about 5 MHz, however we observed much narrower spectrum features than the laser linewidth. In addition, the use of traveling-wave schemes requires that the group-velocity mismatch between two optical waves in either port should be less than the signal modulation period to reduce the temporal walk-off and to achieve effective photon-phonon coupling.

$$(n_{g,A} - n_{g,B})L < 2\pi c/(\omega_0 n_{g,A}), \quad (1)$$

where $n_{g,j}$ represents the group index in waveguide j, L is the length of waveguides, c is the speed of light, and ω_0 indicates the resonant frequency of a PPER system. The group index and effective refractive index of the waveguide are calculated from its geometry as in Supplementary Fig. 1 using 2-D vector-field mode solver.

As seen in Supplementary Fig. 1c, the group index variance of the optical waveguides ($950 \times 220\ \mathrm{nm}^2$) is less than 0.2 % over 1.4 μm to 1.7 μm while the effective refractive index changes by about 9%. This result indicates that the information transfer is largely insensitive over wide range of wavelength with the typical length-scale of the optical waveguides. From these results, we believe that even incoherent light sources (e.g. light emitting diode) can be used which could be a tremendous asset of the PPER system for practical applications in optical signal processing.

Supplementary Note 2

Power handling capability.

We theoretically predict the power handling capability of the silicon waveguide using its propagation loss. In this article, we consider the propagation loss contributed by linear absorption (material absorption and scattering effects) and nonlinear absorption (two photon absorption (TPA) and TPA induced free-carrier absorption) which has been well studied in silicon[2,3]. Then the total absorption coefficient, $\boldsymbol{\alpha}$, can be given by

$$\alpha = \alpha_o + \beta_{\mathrm{TPA}} \frac{P}{A} + \sigma \Delta \mathrm{N} \left(\frac{P}{A}\right)^2, \quad (2)$$

where α_o and β_{TPA} represent the linear absorption and TPA coefficients, respectively. P is the internal optical power in the waveguide, and A is the effective mode area of silicon waveguide. σ represents the free-carrier absorption cross-section, and $\Delta\mathrm{N} = \tau\beta_{\mathrm{TPA}}/2h\nu$ is the free-carrier number density in steady state conditions. τ is the free-carrier lifetime in silicon waveguide, and $h\nu = 1.28 \times 10^{-19}$ [J] is the photon energy of the pump light at 1550 nm. The values of $\beta_{\mathrm{TPA}} = 0.5 \times 10^{-11}$ [m W^{-1}] and $\sigma =$

1.45×10^{-21} [m^2] in silicon are well studied[2,3]. The effective mode area, $A = 1.3 \times 10^{-13}$ [m^2], is numerically solved using COMSOL multi-physics simulations using the physical size of silicon waveguide of $950 \times 220\ \text{nm}^2$. We measured the free-carrier lifetime of $\tau = 2$ ns in our silicon waveguide with a pump-probe experiment, and the linear absorption coefficient, $\alpha_o = 1$ [dB cm^{-1}] = 23 [m^{-1}], is extracted from a cut-back measurement. Using the parameters above, the total absorption coefficient of Eq. S1a in the silicon waveguide can be rewritten as,

$$\alpha = 23 + 38.46 \times P + 3352 \times P^2\ \ [\text{m}^{-1}]. \tag{3}$$

In this article, we define the power handling capability of the silicon waveguide as the power yielding the total absorption coefficient of $\alpha = 3$ [dB cm^{-1}] = 69 [m^{-1}], which was chosen to be consistent with the length-scale of propagation on silicon chips. Hence, the internal optical power of $P = 110$ mW is the power handling of our silicon waveguide. This power in the silicon waveguides can be easily achievable with better fiber-to-waveguide coupling method.

Supplementary Note 3

Temporal coupled mode theory.

The analytical expression of the photon-phonon emitting-receiving functionalities can be achieved using temporal coupled-mode theory[4,5]. As discussed in the main context, we consider two pump fields $\boldsymbol{E}_{\mathbf{1}}^{\boldsymbol{a}}(\omega_1)$ and $\boldsymbol{E}_{\mathbf{2}}^{\boldsymbol{a}}(\omega_2)$ in Wg-A and probe as well as signal fields $\boldsymbol{E}_{\mathbf{3}}^{\boldsymbol{b}}(\omega_3)$ and $\boldsymbol{E}_{\mathbf{s}}^{\boldsymbol{b}}(\omega_s)$ in Wg-B. Here, $\boldsymbol{E}_{\boldsymbol{j}}^{\boldsymbol{a}}(\omega_j, t) \equiv \boldsymbol{h}_{\boldsymbol{a}}(x,y)A_j(z)e^{i(k_j z - \omega_j t)}$ and $\boldsymbol{E}_{\boldsymbol{j}}^{\boldsymbol{b}}(\omega_j) \equiv \boldsymbol{h}_{\boldsymbol{b}}(x,y)B_j(z)e^{i(k_j z - \omega_j t)}$, where $\boldsymbol{h}_{\boldsymbol{a}}$ ($\boldsymbol{h}_{\boldsymbol{b}}$) represents the field mode distribution in Wg-A (Wg-B), and A_j and B_j are power-normalized mode amplitudes such that $|A_j|^2$ ($|B_j|^2$) is the modal power, P_j^a (P_j^b) carried in Wg-A (Wg-B).

By the existence of optical fields in waveguide, the optical forces mediated by electrostriction and radiation pressure can yield elastic displacement within the silicon core. The optical force induced by an optical wave $\boldsymbol{E}_{\boldsymbol{j}}^{\boldsymbol{a}}(\omega_j)$ can be written as, $\mathbf{f}_{\boldsymbol{a}} = \mathbf{f}_{\boldsymbol{n}}^{\boldsymbol{a}}(x,y)\,|A_j|^2 = \mathbf{f}_{\boldsymbol{n}}^{\boldsymbol{a}}(x,y)\,P_j^a$, where $\mathbf{f}_{\boldsymbol{n}}^{\boldsymbol{a}}(x,y)$ is the force density normalized to the modal power in Wg-A under continuous-wave excitation. The optical force density distribution induced by two optical fields ($\boldsymbol{E}_{\mathbf{1}}^{\boldsymbol{a}}(\omega_1)$ and $\boldsymbol{E}_{\mathbf{2}}^{\boldsymbol{a}}(\omega_2)$) in Wg-A consists of a constant dc term and an oscillating term as, $\mathbf{f_a} = \mathbf{f_{DC}} + \mathbf{f_{AC}} = \mathbf{f}_{\boldsymbol{n}}^{\boldsymbol{a}}(x,y)(|A_1|^2 + |A_2|^2) + \mathbf{f}_{\boldsymbol{n}}^{\boldsymbol{a}}(x,y)2A_1A_2^* e^{i(Kz - \Omega t)}$, where $\Omega = (\omega_1 - \omega_2)$ is the beating frequency and $\mathbf{K} = \mathbf{k}_1 - \mathbf{k}_2$ is the phonon wave-vector. Note that we ignore the phase difference between two optical fields.

The oscillating optical force of $\mathbf{f}_{\boldsymbol{n}}^{\boldsymbol{a}}(x,y)2A_1A_2^* e^{-i\Omega t}$ yields elastic displacement field, $\boldsymbol{u}_{\boldsymbol{a}} = \boldsymbol{e}_{\boldsymbol{a}}(x,y)c_a(t) = \boldsymbol{e}_{\boldsymbol{a}}(x,y)C_a e^{-i\Omega t}$, in Wg-A. Note that the optical cross-talk between Wg-A and Wg-B is negligible, but phonons can transfer to Wg-B through the central PnC region, creating phononic defect

mode in Wg-B, $\boldsymbol{u_b} = \boldsymbol{e_b}(x,y)c_b(t) = \boldsymbol{e_b}(x,y)C_b e^{-i\Omega t}$. In the limit of small signal amplitudes, B_s, the phonon dynamics at any position (z) along the length of the waveguides can be expressed as,

$$\frac{dc_a(t)}{dt} = -\left(i\Omega_0 + \frac{1}{\tau_{\text{net}}}\right)c_a(t) + i\mu c_b(t) + \eta(t)A_1A_2^*, \tag{4}$$

$$\frac{dc_b(t)}{dt} = -\left(i\Omega_0 + \frac{1}{\tau_{\text{net}}}\right)c_b(t) + i\mu c_a(t), \tag{5}$$

using temporal coupled-mode theory[5,4]. Here, Ω_0 is the natural frequency of the uncoupled phonon modes, μ represents the phononic modal coupling rate, and τ_{net}^{-1} is the net decay rate of phonon mode, which is related to the external decay (τ_e^{-1}) through either side of PnC cladding and internal (τ_o^{-1}) decay rates as $\tau_{\text{net}}^{-1} = \tau_e^{-1} + \tau_o^{-1}$. Above, $\eta(t)A_1A_2^*$ is the driving term, of the form $\eta(t) = \eta_o \exp(-i\Omega t)$. From Ref. [6], the coupling amplitude, η_o, becomes $\eta_o = \langle \mathbf{f}_{\boldsymbol{n}}^{\boldsymbol{a}}, \boldsymbol{e_a}\rangle \times \langle \boldsymbol{e_a}, \rho\boldsymbol{e_a}\rangle^{-1}(\Omega_o)^{-1}$, where $\langle \boldsymbol{X}, \boldsymbol{Y}\rangle \equiv \int \boldsymbol{X}^* \cdot \boldsymbol{Y} dS$ over the waveguide cross-section and $\rho(x,y)$ is the mass density of the elastic medium. Solutions of Eq. S2a and Eq. S2b yield a set of hybridised (symmetric and anti-symmetric) phonon modes with wave amplitudes $C_a = i\eta_o(\Omega - \Omega_o + i/\tau_{\text{net}})A_1A_2^*\,[\Gamma_-(\Omega)\,\Gamma_+(\Omega)]^{-1}$, and $C_b = i\mu\eta_o A_1A_2^*\,[\Gamma_-(\Omega)\,\Gamma_+(\Omega)]^{-1}$, where $\Gamma_\pm(\Omega) \equiv [\Omega - (\Omega_0 \pm \mu) + i/\tau_{\text{net}}]$. Hence, optical wave-mixing in Wg-A drives an elastic displacement field ($\boldsymbol{u_b(t)}$) in Wg-B.

Now we consider the impact of elastic deformation induced by the phonon field on optical waves propagating in Wg-B. Since light guided in the core of the waveguide produces a force distribution within the core of the waveguide, any elastic displacements will perform work against these optical forces, changing the energy of the guided electromagnetic fields. Using the principle of virtual work, the change in guided electromagnetic energy per unit length in Wg-B produced by a small elastic deformation of amplitude $\boldsymbol{\delta u_b}$ can be expressed as $\delta U_{\text{ME}}/L = \langle \boldsymbol{\delta u_b}, \mathbf{f}^{\boldsymbol{b}}\rangle = \langle \boldsymbol{e_b}, \mathbf{f}_{\boldsymbol{n}}^{\boldsymbol{b}}\rangle \delta c_b P_3^b$. Following Refs. [1,7], one can show that this change in electromagnetic energy is equivalent $\delta U_{\text{EM}} = (P_3^b/\omega_3)\,\delta\phi$ where $\delta\phi$ represents the phase induced by variation in the mechanical degree of freedom δc_b. Hence, the phase change per unit length imparted on wave $\tilde{B}_3$ by elastic wave displacement, $\boldsymbol{u_b(t)}$, can be expressed as $\delta\phi/L = (1/L)(\delta\phi/\delta c_b)C_b = \langle \boldsymbol{e_b}, \mathbf{f}_{\boldsymbol{n}}^{\boldsymbol{b}}\rangle C_b\omega_3$. Using this result, we have the phonon mediated coherent coupling from Wg-A to Wg-B, $\gamma_{a\to b}(\Omega)$, as

$$\gamma_{a\to b}(\Omega) = \left[\frac{\omega_3\tau_{\text{net}}}{2\Omega_0}\,\frac{\langle \mathbf{f}_{\boldsymbol{n}}^{\boldsymbol{a}}, \boldsymbol{e_a}\rangle\,\langle \boldsymbol{e_b}, \mathbf{f}_{\boldsymbol{n}}^{\boldsymbol{b}}\rangle}{\langle \boldsymbol{e_a}, \rho\boldsymbol{e_a}\rangle}\,\frac{2\mu/\tau_{\text{net}}}{\left[\Omega - \Omega_0 - \sqrt{\mu^2 - 1/\tau_{\text{net}}{}^2}\right]\left[\Omega - \Omega_0 + \sqrt{\mu^2 - 1/\tau_{\text{net}}{}^2}\right]}\right]. \tag{6}$$

Then phonon mediated signal field change per unit length in Wg-B can be rewritten as,

$$\frac{\partial B_S}{\partial z} = i[|\gamma_{a\to b}(\Omega)]A_1^*A_2B_3 \tag{7}$$

In low gain regime, the output signal power, P_s^b, is given by $P_s^b = |\gamma_{a\to b}(\Omega)|^2 P_1^a\, P_2^a\, P_3^b\, L^2$.

Supplementary Note 4

Spectrum of PPER responses with enhanced conversion efficiency.

We repeated the experimental measurement of the response of a PPER system with higher internal optical power to enhance the conversion efficiency. The pump and probe beams are coupled into and out of the PPER system using grating couplers having the fiber-to-waveguide coupling efficiency of 18% per each coupler. The estimated pump and probe powers internal to the waveguides are $72\ \mathrm{mW}$ and $30\ \mathrm{mW}$, respectively. Supplementary Fig. 2 shows the measured RF power from the heterodyne measurement. The peak RF power is -15 dBm corresponding to the signal conversion efficiency of 1%. Note that, with the 10 times higher internal pump power, the conversion efficiency increases by a factor of 100 compared to the data in Fig. 3 as signal power increases quadratically with pump power ($\propto P_1^a P_2^a$).

Supplementary Note 5

Spectra of photon-phonon emitter-receiver responses over the stopband frequency range.

We also investigate the responses of the photon-phonon emitter-receiver systems over the stopband frequency range (2.6 – 4.5 GHz, yellow region in Supplementary Fig. 3). Note that this stopband frequency range in Supplementary Fig. 3 matches well with the fundamental phononic stopband from the computed phononic dispersion curves in Fig. 2g. The dual channel PnC-BAM waveguides for $\mathrm{W_o} = 5.7\ \mu\mathrm{m}$ and $\mathrm{W_o} = 5.2\ \mu\mathrm{m}$ are tested under identical experimental conditions. As seen in Supplementary Fig. 3, the normalized RF power for $\mathrm{W_o} = 5.7\ \mu\mathrm{m}$ ($\mathrm{W_o} = 5.2\ \mu\mathrm{m}$) shows a strong peak at 2.93 GHz (3.18 GHz) and flat response over the stopband range. The response for $\mathrm{W_o} = 5.7\ \mu\mathrm{m}$ displays a narrow feature at 4.44 GHz which is a higher order resonant mode. Several signatures produced by complicated phononic modes are also shown at around 3.7 GHz for $\mathrm{W_o} = 5.7\ \mu\mathrm{m}$, but not for $\mathrm{W_o} = 5.2\ \mu\mathrm{m}$, indicating that these complicated features can be removed by engineering the phononic supermodes and the PnC structures.

Supplementary references

1. Rakich, P., Reinke, C., Camacho, R., Davids, P. & Wang, Z. Giant Enhancement of Stimulated Brillouin Scattering in the Subwavelength Limit. *Phys. Rev. X* **2,** 011008 (2012).
2. Lin, Q., Painter, O. J. & Agrawal, G. P. Nonlinear optical phenomena in silicon waveguides: modeling and applications. *Opt. Express* **15,** 16604–16644 (2007).
3. Lin, Q. *et al.* Dispersion of silicon nonlinearities in the near infrared region. *Appl. Phys. Lett.* **91,** 021111 (2007).
4. Haus H A. *Waves And Fields In Optoelectronics*. (Prentice-Hall, 1984).
5. Little, B. E., Chu, S. T., Haus, H. a., Foresi, J. & Laine, J.-P. Microring resonator channel dropping filters. *J. Light. Technol.* **15,** 998–1005 (1997).
6. Qiu, W. *et al.* Stimulated Brillouin scattering in nanoscale silicon step-index waveguides: a general framework of selection rules and calculating SBS gain. *Opt. Express* **21,** 31402–31419 (2013).
7. Rakich, P. T., Popović, M. a & Wang, Z. General treatment of optical forces and potentials in mechanically variable photonic systems. *Opt. Express* **17,** 18116–18135 (2009).
